# Chitosan–Collagen Electrospun Nanofibers Loaded with Curcumin as Wound-Healing Patches

**DOI:** 10.3390/polym15132931

**Published:** 2023-07-02

**Authors:** Maila Castellano, Andrea Dodero, Sonia Scarfi, Serena Mirata, Marina Pozzolini, Eleonora Tassara, Alina Sionkowska, Katarzyna Adamiak, Marina Alloisio, Silvia Vicini

**Affiliations:** 1 Department of Chemistry and Industrial Chemistry, University of Genoa, Via Dodecaneso 31, 16146 Genoa, Italy; maila.castellano@unige.it (M.C.); marina.alloisio@unige.it (M.A.); silvia.vicini@unige.it (S.V.); 2 Adolphe Merkle Institute, University of Fribourg, Chemin des Verdiers 4, 1700 Fribourg, Switzerland; 3 Department of Earth, Environmental and Life Sciences, University of Genova, 16132 Genoa, Italy; soniascarfi@unige.it (S.S.); serena.mirata@edu.unige.it (S.M.); marina.pozzolini@unige.it (M.P.); eleonora.tassara@edu.unige.it (E.T.); 4 Inter-University Center for the Promotion of the 3Rs Principles in Teaching & Research (Centro 3R), 56122 Pisa, Italy; 5 Department of Chemistry of Biomaterials and Cosmetics, Nicolaus Copernicus University, 87100 Toruń, Poland; alinas@umk.pl (A.S.); kadamiak@wellu.eu (K.A.)

**Keywords:** chitosan, collagen, curcumin, electrospun nanofibers, wound-healing patches

## Abstract

Composite chitosan–collagen nanofibrous mats embedded with curcumin were prepared via a single-step electrospinning procedure and explored as wound-healing patches with superior biological activity. A mild crosslinking protocol consisting of a short exposure to ammonia vapor and UV radiation was developed to ensure proper stability in physiological-like conditions without affecting the intrinsic biocompatibility of chitosan and collagen. The fabricated composite patches displayed a highly porous, homogeneous nanostructure consisting of fibers with an average diameter of 200 nm, thermal stability up to 200 °C, mechanical features able to ensure protection and support to the new tissues, and water-related properties in the ideal range to allow exudate removal and gas exchange. The release kinetic studies carried out in a simulated physiological environment demonstrated that curcumin release was sustained for 72 h when the mats are crosslinked hence providing prolonged bioactivity reflected by the displayed antioxidant properties. Remarkably, combining chitosan and collagen not only ensures prolonged stability and optimal physical–chemical properties but also allows for better-promoting cell adhesion and proliferation and enhanced anti-bacteriostatic capabilities with the addition of curcumin, owing to its beneficial anti-inflammatory effect, ameliorating the attachment and survival/proliferation rates of keratinocytes and fibroblasts to the fabricated patches.

## 1. Introduction

Skin chronic diseases and traumatic damage are great healthcare issues, with wound management representing a significant financial burden [1]. Up to now, the gold standard in wound treatments consists of autologous skin grafting, whose use is, unfortunately, strongly limited by the dimensions of the damaged area [2]. Allogeneic or xenogeneic skin grafting is commonly employed despite the high risks of immune rejection and disease transmission [3]. As a consequence, scientists are working in close contact with medical doctors to develop healing patches to protect the wound and promote tissue repair and regeneration [4]. As a matter of fact, the dressings commonly used in clinical practice (i.e., gauze, sterilized absorbent cotton, and bandages) offer only physical protection and have very limited benefits for wound healing and infection prevention. Thus, developing modern, advanced wound patches with superior properties is urgent [5,6]. In this context, electrospinning technology is acquiring increasing interest due to its simplicity, cost-effectiveness, and versatility in fabricating nanostructured mats that reproduce the morphological characteristics of the skin extracellular matrix (ECM) [7]. As such, these nanofibrous meshes show great capabilities in supporting cell adhesion, migration, growth, and differentiation, as well as angiogenesis, which are all vital processes for the occurrence of an effective wound-healing process [8]. Another advantage of electrospinning is the possibility of adding nanofibers with several substances, ranging from nanoparticles to bioactive molecules, that can embed the final product with enhanced biological performances [9,10]. In recent decades, electrospun wound-healing patches have been mostly prepared using synthetic, biocompatible polymers due to their high stability in physiological conditions, superior mechanical properties, and ease of processing [11]. However, such electrospun patches cannot completely fulfil the need to obtain efficient wound-healing systems due to their synthetic nature and the use of harsh solvents during their processing. To solve this problem, natural polymers (e.g., alginate, chitosan, hyaluronic acid, collagen, etc.) have recently started to be used as a promising alternative owing to their higher affinity with biological tissues [12,13,14]. However, electrospinning these natural materials is usually more difficult and the obtained electrospun mats often display limited stability in the physiological environment, thus requiring crosslinking treatments with hazardous substances that may reduce their biocompatibility [15]. Among others, chitosan and collagen have shown promising capabilities in biomedical applications [16,17,18,19]. Chitosan is a polysaccharide derived from chitin, the second most abundant polymer in nature, showing excellent properties in terms of biocompatibility, biodegradability, bioactivity, antibacterial effects, non-toxicity, and good adsorption properties, thus being an extremely suitable and essential biomaterial drawing a great deal of industrial attention as a future alternative to synthetic polymers [20,21]. Collagen is the most abundant structural protein in the extracellular matrix of the various connective tissues in the body (i.e., skin, bones, ligaments, tendons, and cartilage), and it is a crucial component of the wound-healing process. Collagen acts as a natural structural substrate for new tissue formation and growth, playing an essential role in all phases of wound healing, including hemostasis, inflammation, proliferation, and remodeling [22,23].

With these premises, in this work, composite electrospun membranes made of chitosan and collagen were fabricated in a single-step procedure by using poly(ethylene oxide), a water-soluble synthetic polymer, as a co-spinning agent and embedded with curcumin, a potent, natural active molecule with important biological properties. Curcumin is the principal curcuminoid of the turmeric rhizome (*Curcuma longa*) and was widely used in Ayurveda, Siddha, and traditional Chinese medicine for centuries due to its several therapeutic properties (i.e., analgesic, anti-inflammatory, antioxidant, antiseptic, and anti-carcinogenic properties). Recently there has been increasing attention on the use of curcumin for regenerative medicine applications focused on the wound-healing process [24]. The fabricated mats were subjected to a mild physical crosslinking treatment to improve their stability in physiological conditions and then characterized in terms of their morphological, thermal, mechanical, water-related, and release properties. Additionally, their capability to promote cell adhesion and proliferation was fully investigated, along with their antioxidant properties and their ability to reduce bacterial adhesion. We hypothesized that combining collagen, which possesses strong biological activities, and chitosan, which ensures superior mechanical stability and support, with the bioactive properties of curcumin would provide a novel, efficient platform for wound healing and tissue regeneration.

## 2. Materials and Methods

### 2.1. Materials

Low-molecular-weight chitosan (CS) (20–300 cP, 1 wt.% in 1% acetic acid, 25 °C, 75–85% deacetylated) was obtained from Sigma-Aldrich (St. Louis, MO, USA) and used without further purification. Collagen was purchased from WellU sp.z.o.o, Gdynia, Poland. It was obtained by collagen isolation from *silver carp* skin. The yield of the collagenous proteins extraction process is 95%. The skin fragments were removed manually and washed with chilled tap water to remove the adhering tissues. In the next stage, the material was treated with a 3% hydrogen peroxide water solution, the residues of which were rinsed off afterwards. The purified skin was placed in an acetic acid solution for 3 days to extract the collagenous proteins. The obtained solution was pressed through material, allowing collagen separation. The samples were then placed in polyethylene bags and stored at T = −25 °C until used. The collagen solution was dialyzed against distilled water for 2 days and then lyophilized. Poly(ethylene oxide) (PEO) with an average ${\overset{-}{M}}_{v}=900\;kg/mol$, Triton™ X-100, curcumin from *Curcuma longa* (Turmeric) with purity (HPLC) ≥ 65%, ammonium hydroxide (NH_4_OH), absolute ethanol (EtOH), methanol (MeOH), sodium phosphate monobasic (NaH_2_PO_4_), sodium phosphate dibasic (Na_2_HPO_4_), sodium chloride (NaCl), and ascorbic acid were purchased from Sigma Aldrich. 2,2-diphenyl-1-picrylhydrazyl (DPPH) was obtained from Millipore (Burlington, MA, USA). The human keratinocyte HaCaT cell line and mouse fibroblast L929 cell line were obtained from LGC Standards (London, UK). Dulbecco’s Modified Eagle’s Medium (DMEM), glutamine, and Fetal Bovine Serum (FBS) were purchased from Euroclone (Lima, Peru).

### 2.2. Methods

#### 2.2.1. Solution Preparation

Chitosan powder was dissolved under magnetic stirring at room temperature using a 10% *v*/*v* acetic acid water solution to obtain a polymer concentration of 2.5% *w*/*v*. Collagen powder was dissolved under magnetic stirring at T = 10 °C using a 10% *v*/*v* acetic acid water solution to obtain a polymer concentration of 2.5% *w*/*v*. PEO powder was dissolved under magnetic stirring at room temperature using a 10% *v*/*v* acetic acid water solution to obtain a polymer concentration of 2.5% *w*/*v*. After complete solubilization, chitosan or collagen solutions were mixed with the PEO solution in a 1:1 ratio before adding 1% *w*/*v* Triton. Chitosan–collagen solutions were obtained by mixing the chitosan, collagen, and PEO solutions in a 1:1:2 ratio before adding 1% *w*/*v* Triton. The mixtures were magnetic stirred at T = 10 °C for a further 24 h and finally kept at 4 °C to prevent polymer degradation. For the preparation of curcumin-loaded mats, curcumin powder was added directly to the polymer solution at a 1% *w*/*w* concentration with respect to the polymer weight.

#### 2.2.2. Rheological Characterization

The prepared solutions were characterized by a rotational rheometer (Anton Paar MCR-301) equipped with a Peltier heating system and a solvent trap kit. All measurements were performed at T = 25 °C (samples were thermally equilibrated for 300 s) using a cone–plate geometry with a diameter of 50 mm, a truncation angle of 0.989°, and a gap of 99 μm (CP50-1). Steady-state viscosity curves were evaluated in the 0.01–1000 1/s shear rate range. Amplitude sweeps were carried out at 1 Hz frequency in the oscillatory strain range of 0.001–10%. Frequency sweeps were performed at 0.1% oscillatory strain in the oscillatory frequency range of 0.01–10 Hz.

#### 2.2.3. Electrospinning and Crosslinking

The polymer solutions were electrospun using a Doxa Microfluidics^®^ Professional Electrospinning Machine equipped with a temperature and humidity control system. In a typical procedure, 10 mL of polymer solution was electrospun into an aluminum flat collector using a 10 mL glass syringe connected to a needle with an inner diameter of 0.6 mm. The processing parameters were selected using a high-resolution digital camera to visualize the Taylor cone and consisted of a flow rate of 1 mL/h, an applied voltage of 25 kV, a needle-collector distance of 15 cm, a temperature of 25 °C, and a relative humidity of 30%. Electrospun mats were first kept under vacuum at 40 °C overnight to remove any residual solvent and then subjected to a crosslinking treatment to increase their stability in physiological conditions. Specifically, the mats were first treated with NH^4+^ vapor for 4 h and then subjected to a 10 min irradiation with UV light in a Rayonet Photochemical Chamber Reactor, Model RPR-200 (254 nm, 35 W, 16,000 µW/cm^2^). No further treatments were carried out. 

#### 2.2.4. Field Emission Scanning Electron Microscopy 

Morphological investigation of electrospun mats was carried out using a Field-Emission Scanning Electron Microscope (FESEM) ZEISS SUPRA 40 VP operating in InLens mode configuration. Samples were thinly sputter-coated with carbon using a Polaron E5100 sputter coater to obtain good conductivity. Size evaluation of fibers from FESEM images was performed with the open-source software ImageJ. 

#### 2.2.5. Thermogravimetric Analysis and Differential Scanning Calorimetry

Thermal degradation profiles of the polymers, curcumin, and electrospun membranes were evaluated by means of ThermoGravimetric Analysis (TGA) carried out with a Mettler-Toledo TGA/DCS1 STARe System. A dynamic mode operating in the range from 30 to 700 °C under an N_2_ atmosphere (gas flow of 80 mL/min) and from 700 to 900 °C under an O_2_ atmosphere (gas flow of 80 mL/min) with a heating rate of 10 °C/min was employed. Thermal transitions of the polymers, curcumin, and electrospun membranes were investigated via Differential Scanning Calorimetry (DSC) using a Mettler Toledo DSC1 STARe instrument. Measurements were performed under an N_2_ atmosphere (10 mL/min) in the temperature range from 0 to 150 °C with a heating rate of 20 °C/min. 

#### 2.2.6. Fourier-Transform Infrared Spectroscopy

Fourier-Transform Infrared Spectroscopy (FTIR) was carried out on the polymers, curcumin, and electrospun membranes by means of a Bruker Vertex 70 instrument operating in ATR mode. Each spectrum was collected in the wavenumber range of 400–4000 1/cm at a resolution of 4 1/cm.

#### 2.2.7. Dynamic Mechanical Analysis

Dynamic Mechanical Analysis (DMA) was performed on the electrospun mats after crosslinking in extensional configuration by using a rotational rheometer Anton Paar MCR-301 equipped with a universal extensional fixture geometry (UXF) and a CDT-450 chamber. Measurements were performed in duplicate on rectangular specimens (40 mm × 10 mm) at T = 37 °C. Amplitude sweeps were carried out at 1 Hz frequency in the oscillatory stress range of 0.01–1 MPa. Frequency sweeps were performed at 0.1 MPa oscillatory stress in the oscillatory frequency range of 0.01–10 Hz.

#### 2.2.8. Stability and Water-Related Properties

The stability of the crosslinked electrospun membranes was assessed by immersing them in PBS at T = 37 °C for 2 weeks. Mat weight loss (Δm) was evaluated as:

(1) $$∆m\;(\%)=\frac{{m_{i}-m}_{f}}{m_{i}}\times 100$$

where m_i_ and m_f_ are the initial and the final mass of the mats, respectively. The water contact angle (WCA) of the crosslinked membranes was measured via an Attention Theta Lite optical tensiometer. A small drop of water (i.e., volume = 3 μL) was placed on the sample surface, and both right and left WCA were calculated via the instrument software. Water vapor permeability (WVP) of the crosslinked membranes was assessed by means of a gravimetrical method according to ASTM E96–95. Briefly, circular samples were mounted on measuring cups with a diameter of 9.5 mm which were filled with distilled water up to 2 cm underneath the film. The cups were placed in an environmental chamber at T = 37 °C and RH% = 50 and weighed every hour for a period of 8 h. WVP was calculated as:

(2) $$WVP=\frac{\left(WVTR\cdot d\right)}{\left(A\cdot ∆p\right)}$$

where WPVR is the water vapor transmission rate (g/s), d (m) is the thickness of the sample, A (m^2^) is the surface of the sample that permits the vapor diffusion, and ∆p (Pa) is the partial water vapor pressure difference across the two sides of the sample. The moisture content (MC) of the crosslinked membranes was determined by placing a small piece of each sample at T = 110 °C under a vacuum for 24 h and evaluating the weight loss after drying. MC percentage on a wet basis was expressed as:

(3) $$MC\;(\%)=\frac{m_{i}-m_{f}}{m_{i}}\times 100$$

where m_i_ and m_f_ are the sample mass before and after the drying, respectively.

#### 2.2.9. Curcumin Release Kinetics In Vitro

In vitro release studies of curcumin from pristine and crosslinked electrospun mats were carried out in phosphate-buffered saline (PBS, pH = 7.4) at T = 37 °C to simulate physiological conditions. In a typical experiment, 10 mg of curcumin-loaded membranes were placed in a 1 cm pathlength cuvette filled with 3.5 mL of PBS and sealed. The system was gently shaken using a tilting agitator, and the concentration of the supernatant was periodically monitored through UV-vis spectroscopy for up to 3 days. Electronic absorption spectra were acquired through a UV-1800 spectrophotometer at room temperature in the 200–1000 nm range. The curcumin concentration in the supernatant fraction was calculated at the maximum absorbance from corresponding calibration curves. The cumulative release percentage (R%) was determined as $\frac{m_{r}}{m_{i}}\times 100$ where m_r_ is the curcumin amount released at time t, and m_i_ is the curcumin load in the membrane at time t_0._

#### 2.2.10. Biological Properties

##### DPPH Radical Scavenging Activity

For the quantification of the radical scavenging activity of the different membranes, the protocol of Pozzolini et al. was followed [25]. In detail, mat fragments with an area of 0.9 cm^2^ were immersed in 500 µL of PBS and incubated at room temperature for either 15 min or 60 min. The membranes were removed and then the obtained samples were processed by adding 500 µL of methanol and 250 µL of 0.1 mM DPPH in a methanol solution. A negative control with only DPPH solution and a positive control with ascorbic acid (0.5 mg/mL, final concentration) were also tested. All samples were then incubated for 30 min at room temperature in the dark and subsequently read at 517 nm using a Beckman spectrophotometer (DU 640). In the blank sample, the DPPH solution was substituted with methanol. The antioxidant activity of the samples was measured as the inhibition percentage of DPPH radicals with the following equation:

(4) $$DPPH\;radical\;scavenging\;activity\;(\%)=\frac{\left(A_{0}-A\right)}{A_{0}}\times 100$$

where A and A_0_ are the sample and the negative control absorbance rates, respectively. Data are the means ± S.D. of three independent experiments performed in duplicate.

##### Cell Adhesion and Proliferation

HaCaT and L929 cells were cultured at 37 °C in a humidified, 5% CO_2_ atmosphere in high-glucose DMEM with glutamine, which was supplemented with 10% FBS using penicillin/streptomycin as antibiotics. To evaluate cell adhesion and proliferation, the electrospun membranes were cut into circular discs with a diameter of 6 mm, and following a 30 min sterilization with UVC, they were positioned at the bottom of 96-well plates. Then, both HaCaT and L929 were seeded at 20,000 cells/well onto the membranes, while a standard curve was plated in the 1250–20,000 cells/well range to obtain a linear regression curve to fit the experimental results and extrapolate the number of cells attached to the different membranes. While cell adhesion was evaluated after cells were incubated for 16 h, their proliferation was measured at three different time points, namely 24 h, 72 h, and 120 h; the medium changed every 24 h to remove unattached cells. At the end of the experiments, cell viability was assayed by the MTT test (0.5 mg/mL final concentration) as previously described by Dodero et al. [26], and the absorbance was read at 540 nm by a plate reader (BMG Labtech, Ortenberg, Germany). Data are the means ± S.D. of three independent experiments performed in quadruplicate.

##### Bacterial Adhesion Evaluation

The method reported by Dodero et al. [27] was applied to evaluate the extent of bacterial adhesion on the crosslinked electrospun mats of three different strains of bacteria: the Gram-negative *Escherichia coli* (strain ATTC25404) and *Pseudomonas aeruginosa* (strain ATTC27853) and the Gram-positive *Staphylococcus aureus* (strain ATTC29273). In short, a single colony of each strain was isolated from an LB/agar plate and cultured at 37 °C for 6−8 h in 3 mL of LB medium (10 g/L peptone, 10 g/L sodium chloride, and 5 g/L yeast extract) until reaching the exponential growth phase. The bacterial suspension was centrifuged at 2000× *g* for 10 min, and then the bacterial pellet was washed twice with sterile PBS and resuspended in sterile PBS, obtaining a final density of 10^7^ CFU/mL. The different membranes were cut into 1.5 × 1.5 cm squares, sterilized, soaked in 5 mL of the bacterial suspension or in 5 mL of sterilized PBS as the negative control and shaken at 150 rpm at 37 °C for 5 h. Finally, the membranes were carefully recovered with sterile tweezers and doubly rinsed with sterile PBS to wash away the non-adhering bacterial cells. The number of bacteria adhering to the surface of the membranes was evaluated using a quantitative PCR (qPCR) approach. Bacterial genomic DNA was extracted from each type of electrospun mat with a GeneUP^TM^ gDNA kit (Biotechrabbit), following the manufacturer’s instructions. The DNA was eluted in 100 μL of sterile 50 mmol/L Tris−HCl, pH 8, and 3 μL of it was used for qPCR amplification. The qPCR reactions were conducted in a 15 μL volume containing a 1 × iQ SYBR Green master mix (Bio-Rad Laboratories, Milan Italy) and 0.2 μmol of each specific primer (*Escherichia coli*: F 5′-CATGCCGCGTGTATGAAGAA-3′ and R 5′-CGGGTAACGTCAATGAGCAAA-3′; *Staphylococcus aureus*: F 5′-AGCGTTCGTCCTGCACAAGT-3′ and R 5′-TCATCCTTCGCCTCCCTG-3′; *Pseudomonas aeruginosa*: F 5′-CGAAAGGGCAATACGCAAAG and R 5′-TGTTGGATCTTCAGAACCACTTC). All reactions were performed in triplicate. The thermal cycling conditions were an initial denaturation at 95 °C for 3 min followed by 45 cycles with denaturation at 95 °C for 15 s and annealing and elongation at 60 °C for 60 s. At the end of each elongation step, the fluorescence was measured. The last step was a slow heating (1 °C/s) of the amplified product from 55 to 92 °C in order to generate a melting curve. Data analyses were performed using the DNA Engine Opticon 3 real-time detection system Software (V3.03). Lastly, the total number of bacterial cells on the different membranes was interpolated from a standard curve of DNA derived from 10-fold dilutions of a suspension of each bacteria. Data are the means ± S.D. of three independent experiments.

## 3. Results

### 3.1. Physical–Chemical Characterization of Electrospun Membranes

**Figure 1:**
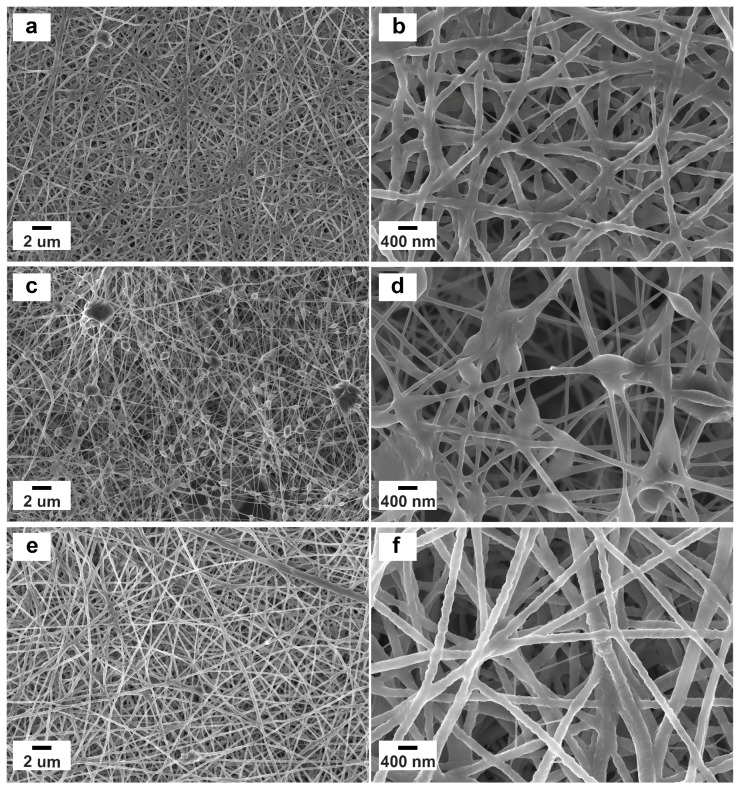
Nanofibrous structure after the crosslinking treatment for (**a**,**b**) chitosan–curcumin electrospun mat, (**c**,**d**) collagen–curcumin electrospun mat, and (**e**,**f**) chitosan–collagen–curcumin electrospun mat. Samples show an overall homogenous morphology, with the composite sample presenting the largest and most regular nanofibers. The absence of observable curcumin crystals indicates their effective encapsulation within the polymeric nanofibers.

The starting solution rheological properties (i.e., viscosity and viscoelasticity) highly influence the electrospinning process. These are crucial to ensure good stability and stretchability of the polymer jet during flight time, to prevent the formation of nanobeads/nanodroplets, and to modulate the dimension of the resulting nanofibers [28,29]. Previous studies on biopolymer mixtures showed a viscosity value of around 10 Pa·s to be ideal for obtaining homogeneous nanometric fibers forming non-woven electrospun membranes [30]. Steady-state viscosity curves are reported in Figure S1 for chitosan-based, collagen-based, and chitosan–collagen-based solutions. The three samples presented a viscosity in the optimal range for the electrospinning process and displayed an initial plateau followed by a well-defined shear-thinning behavior (i.e., viscosity decreased with increasing shear rate) due to the progressive alignment of the polymer chains. The good agreement of the experimental curves with the Carreau–Yasuda model [31] indicates good quality and homogeneity of the mixtures. The viscoelastic properties of the solutions were evaluated via oscillatory rheology (i.e., amplitude sweep and frequency sweep tests) and are shown in Figure S2. The three mixtures were characterized by a liquid-like behavior in the investigated frequency range, and a crossover between the moduli was observed only for the chitosan–collagen-based solution, which is likewise due to the establishment of hydrogen bonding between the two polymers that increases the mixture elastic response [32]. Adding curcumin powder to such solutions turned them a bright yellow color but did not modify their properties. The electrospun membranes obtained from such solutions were subjected to a stabilizing/physical crosslinking procedure by exposing them to NH^4+^ vapor first and then to UV radiation [33,34]. The obtained mats were characterized in detail in both their pristine and crosslinked forms in order to evaluate their potential as biomedical and pharmaceutical products. Figure 1 and Figure S3 show the FESEM micrographs and the fiber dimension distribution for the samples containing curcumin after crosslinking, respectively. Despite a nanofibrous structure being observed for all mats, some differences were found depending on the sample composition.

The chitosan–curcumin membrane showed homogenous, densely-packed nanofibers with an average diameter of 182 ± 50 nm (Figure 1a,b). On the contrary, the collagen–curcumin membrane displays the smallest (i.e., 112 ± 46 nm) and least homogeneous nanofibers characterized by several nanobeads (Figure 1c,d). Such a difference is attributable to the lower solubility of collagen compared to chitosan in aqueous environments. Remarkably, the composite mat presents the largest (i.e., 196 ± 46 nm) and the most regular nanofibers, forming a highly porous, defect-free structure (Figure 1e,f) and representing the ideal platform for wound-healing patches. Curcumin crystals could not be clearly observed in all cases, thus suggesting their efficient encapsulation within the nanofibers.

**Figure 2:**
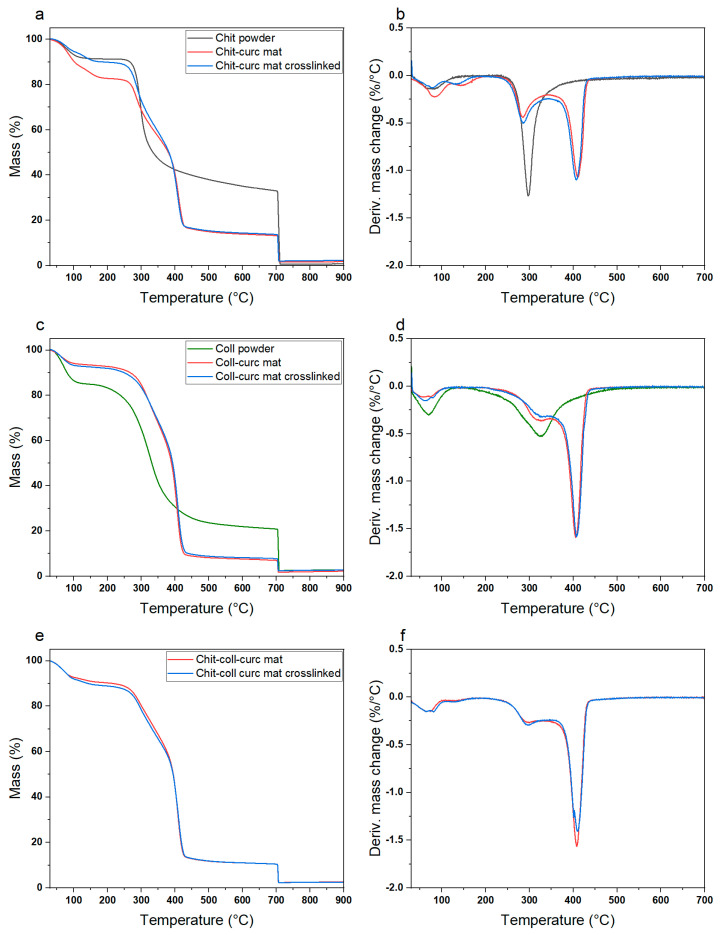
TGA and DTGA profiles for (**a**,**b**) chitosan-based electrospun mat, (**c**,**d**) collagen-based electrospun mat, and (**e**,**f**) chitosan–collagen-based electrospun mat. The peaks observed in the DTGA profiles are indicative of the different degradation processes occurring with increasing temperature (i.e., vaporization of the residual humidity at T < 100 °C, degradation of chitosan or collagen at T~300 °C, degradation of PEO at T~400 °C). Notably, the small changes observed between pristine and crosslinked mats indicate the absence of significant physical–chemical changes occurring during the crosslinking treatment.

Thermogravimetric analysis and differential scanning calorimetry were performed to assess the sample composition and thermal stability. Figure 2 reports the TGA and DTGA profiles for the chitosan-based, collagen-based, and chitosan–collagen-based samples. All samples (i.e., biopolymer powders and electrospun mats before and after crosslinking) presented a weight loss at T < 150 °C that is ascribable to the evaporation of residual humidity and bound water. Considering the electrospun mats, these were characterized by an initial degradation step at T~300 °C, which is associated with the biopolymer fraction (i.e., chitosan, collagen, or both), and a second one at T~400 °C, which is related to the PEO (Figure S4) used as the co-spinning agent and present in the nanofibers. As expected, comparing the weight losses of the two degradation steps, the composition of the electrospun mats can be estimated to be 1:1 for the biopolymeric and co-spinning agent parts. It is important to note that the stabilizing crosslinking procedure does not affect the thermal properties of the nanofibrous mats, indicating that no degradation occurs during the exposure to NH^4+^ vapors and UV light. DSC was carried out to further investigate the electrospun membrane composition and thermal properties (Figure S5). PEO powder presented a neat melting phenomenon at T_m_~70 °C, whereas no transition could be discerned for curcumin. Both chitosan and collagen powders showed a broad endothermic peak centered at around 80 °C associated with removing residual humidity in agreement with the TGA data (Figure 2). As expected, pristine mats presented the characteristic peaks of the raw materials, whereas no significant differences were observed for the crosslinked samples.

**Figure 3:**
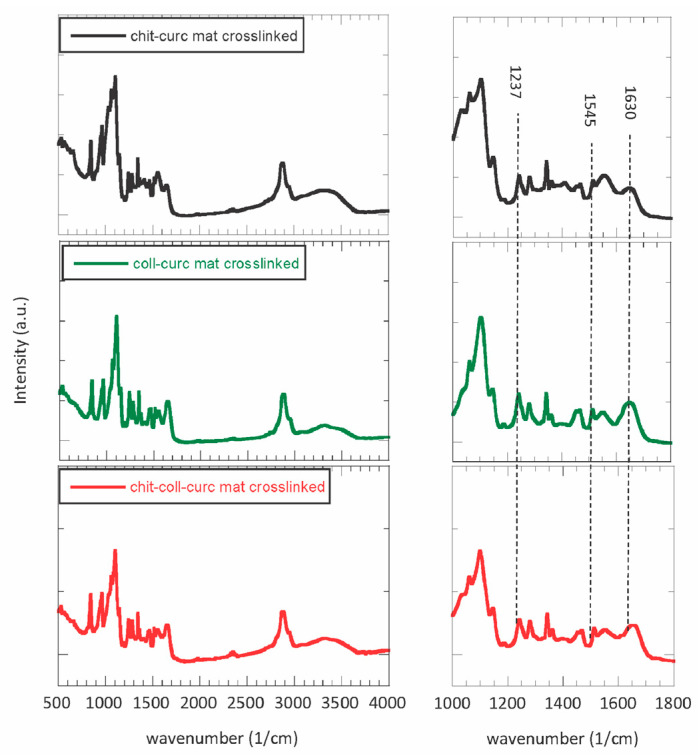
FTIR spectra for chitosan–curcumin crosslinked mat (grey line), collagen–curcumin crosslinked mat (green line), and chitosan–collagen–curcumin mat (red line). All the absorption bands of the raw materials can be observed along with a slight shift in the position of amide I, II, and III bands of collagen, as highlighted in the enlarged spectra on the right. The observed shifts are likewise due to the occurrence of a new linkage between the polymeric chains.

More insights into the interactions occurring between chitosan and collagen were obtained from FTIR spectroscopy. The resultant spectra for the chitosan–curcumin crosslinked mat, collagen–curcumin crosslinked mat, and chitosan–collagen–curcumin crosslinked mat are reported in Figure 3. The spectra of the raw materials and pristine electrospun mats are reported in Figure S6 and S7, respectively. The raw chitosan spectrum depicts characteristic absorption bands at 3350 1/cm attributed to the −OH group, at 2880 1/cm attributed to the -CH_3_ group, at 1650 1/cm attributed to the stretching of the C=O group, at 1570 1/cm attributed to the N-H group bending vibration, at 1420 1/cm attributed to the vibrations of the −OH group of the primary alcoholic group, at 1318 1/cm attributed to the stretching of the C-O-N group, at 1150 1/cm attributed to the glycosidic bond, and at 1030 attributed to the stretching of the C-O group [35]. The raw collagen spectrum mainly presented bands at 1630 1/cm, at 1545 1/cm, and at 1237 1/cm, which are typical of the amide I, II, and III bands, respectively. Additional bands were observed at 3300 1/cm corresponding to the stretching of −OH group, at 2930 1/cm corresponding to the stretching of −CH_3_ group, and at 1454 1/cm corresponding to the stretching of the pyrrolidine rings [36]. Concerning PEO powder, it showed the typical bands at 2885 1/cm assigned to the asymmetric stretching of the methylene group, at 1467 1/cm assigned to the scissoring of the -CH_2_ group, at 1341 1/cm assigned to the asymmetric bending of −CH_2_ group, at 1241 1/cm assigned to the symmetric twisting of the −CH_2_ group, at 1093 1/cm assigned to the C-O-C stretching mode, at 961 1/cm assigned to the asymmetric rocking motion of the −CH_2_ group, and at 840 1/cm assigned to the stretching of the C-O group [37]. Finally, curcumin powder was characterized by the main absorption bands at 3508 1/cm associated with the −OH group, at 1507 1/cm associated with the stretching of the aromatic C-C group, at 1427 1/cm associated with the in-plane deformation of the −OH group, at 1151 1/cm associated with the in-plane deformation of the aromatic −CH group, at 1028 1/cm associated with the out plane deformation of the aromatic −CH group, and at 961 1/cm associated with the bending of the −CH_2_ group [38]. Comparing the FTIR spectra of the raw materials (Figure S6) with those of the pristine electrospun mats (Figure S7), the characteristic bands of the parent molecules can be observed without the onset of additional absorption peaks. However, the chitosan–collagen mats presented a slight shift in the position of amide I, II, and III bands due to the occurrence of a new linkage between the chains containing the chitosan and collagen, either via hydrogen bonding (H-O-H) or through electronic interactions between the cationic chitosan and neighboring anionic collagen [39]. Interestingly, no significant changes were observed for the crosslinked mats (Figure 3) compared to the pristine mats (Figure S7), indicating that the crosslinking protocol does not alter the molecular structure of the biopolymers.

**Figure 4:**
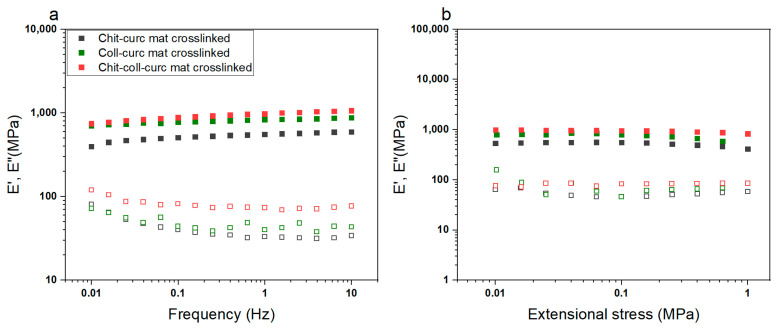
Dynamic mechanical properties of crosslinked electrospun mats in extensional configuration obtained in (**a**) frequency sweep mode and (**b**) stress sweep mode. All samples were within the LVER for the investigated stress range and displayed the typical behavior of solid-like materials with a predominance of the storage modulus over the loss one. Chitosan–collagen–curcumin crosslinked mats were characterized by the highest moduli as a result of the intermolecular interactions between the biopolymers with a consequent increase in the material stiffness.

The mechanical behavior of the crosslinked mats was evaluated via DMTA measurements (i.e., frequency sweep tests and stress sweep tests), and the results are reported in Figure 4. All samples presented similar properties, with an increase of the storage modulus increasing the frequency and the opposite trend for the loss modulus (Figure 4a). Additionally, the mats appeared to be within the linear viscoelastic region (LVER) along the investigated stress range with a not significant decrease of the moduli at high values, most likely due to the disruption of the nanofibrous structure. In terms of values, E’ and E” agree with similar systems and are in the ideal range for wound healing applications similar to those of human skin [40]. Interestingly, the collagen-based mat presented the lowest moduli, the chitosan-based one had an intermediate behavior, and the chitosan–collagen sample exhibited the highest values. Such a result can be ascribed to the intermolecular interactions between chitosan and collagen, which can increase the material stiffness.

Along with an appropriate nanostructure and mechanical properties, electrospun mats with potential uses as wound-healing patches should possess specific water-related properties (i.e., hydrophilicity, vapor permeability, and moisture content) and stability in physiological conditions. 

As shown in Table 1, independent of the composition, all samples presented marked hydrophilicity (i.e., WCA < 90°), thus being suitable for allowing cell viability. Specifically, the collagen-based mat presented a much lower water contact angle than the chitosan one, which agrees with the poor solubility of chitosan in aqueous environments, with the composite sample showing an intermediate behavior. More interestingly, the chitosan–collagen-based mat displayed a much lower water vapor permeability than the single component samples. This finding is associated with the nanofibers’ larger diameter, which is expected to decrease the overall porosity of the nanostructure, thus slowing down the diffusion of water molecules. However, all measured values demonstrated that the mats can provide good gas exchange and exudate removal. Despite such differences, the three samples presented a relatively low moisture content, allowing for safe long-term storage without negatively affecting the material’s physical–chemical properties. Finally, the mat stability in physiological conditions (i.e., PBS buffer solution at pH = 7.4 and T = 37 °C) was assessed. From the data summarized in Table 1 and the SEM micrographs reported in Figure S8, it can be observed that the electrospun collagen mat was almost completely destroyed over a period of 2 weeks. On the contrary, over the same time period, the chitosan-based sample showed a weight loss of around 60% and the composite mat of around 70%. Considering that PEO is still present in the samples (i.e., weight ratio of 50%), the presence of chitosan seems fundamental in stabilizing the mats to avoid their fast dissolution, even if the fibrous nanostructure was mostly lost. 

### 3.2. Curcumin In Vitro Delivery Kinetics

**Figure 5:**
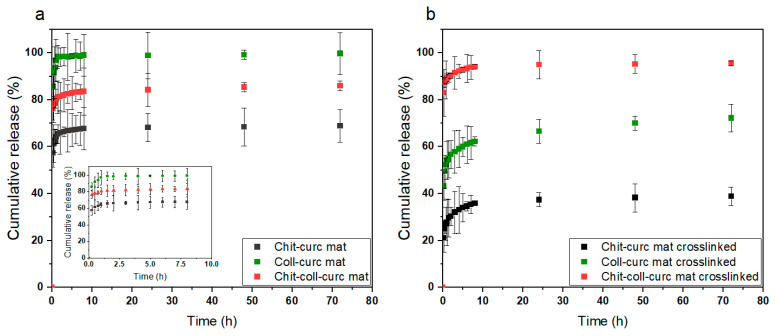
Curcumin release kinetics in vitro for (**a**) pristine electrospun mats and (**b**) crosslinked electrospun mats. Collagen-based mats always presented the highest cumulative release, chitosan-based ones the lowest, and composite patches an intermediate value that agrees with the different stabilities of the samples in physiological conditions. The pristine samples displayed an almost immediate release occurring in the first 5 h, whereas the crosslinked ones were characterized by an initial burst release in the first 10 h followed by a slow release up to 72 h.

Since curcumin is a potent, natural anti-inflammatory substance with marked antioxidant properties, its release kinetics in physiological conditions was evaluated for both pristine and crosslinked samples with the results depicted in Figure 5a and Figure 5b, respectively [41]. In Figure 5a, a rapid release can be observed within the first 5 h (i.e., burst release), reaching 100% for the collagen-based mat, 70% for the chitosan-based one, and around 85% for the double component sample. Such a trend agrees with the previously discussed results and is associated with the different solubility of the polymers in the studied environment. Concerning the crosslinked patches (Figure 5b), a slower and more controlled release kinetic was observed due to their higher stability compared to their pristine counterparts. First, the initial fast release occurred in around 10 h, and then it slowly continued up to 72 h. Second, whereas a 100% release of curcumin was observed for the collagen-based sample, this decreased to only 50% for the chitosan-based mat, with the composite patch showing an intermediate value of 70%. These relatively low values suggest that part of the curcumin is completely embedded in the nanofibers and cannot be released unless the structure fully dissolves. Considering this observed behavior, it is possible to assume that the chitosan–collagen-based crosslinked mat displays an ideal release kinetic for applications targeting tissue regeneration where the controlled release of curcumin is needed to sustain cell viability, while simultaneously providing strong, prolonged antibacterial and antioxidant properties. However, the other mats might still find applications in drug delivery systems where a burst release is required (e.g., trauma wounds). 

### 3.3. Antioxidant Activity, Cell Adhesion, and Survival/Proliferation

Considering the targeted application of the prepared patches in supporting and protecting damaged tissues, such as skin or blood vessels, an anti-inflammatory action would be highly beneficial to accelerate the healing process of the tissues themselves since it is well known that inflammation impairs wound closure and tissue recovery [42]. Antioxidant substances are able to exert anti-inflammatory activity by scavenging of reactive oxygen species (ROS), which have a direct impact on tissue damage and on the signal transduction pathways promoting the inflammatory process itself. Thus, the antioxidant activity of the molecular species released by the membranes in PBS obtained from soaking the mats for 15 or 60 min was evaluated by the DPPH scavenging assay. The results are displayed in Figure S9 and show that, indeed, a significant radical scavenging activity was measurable in the PBS of the three curcumin-embedded membranes as compared to the respective membranes built in the absence of curcumin where no scavenging activity was measured for the observed incubation times. Thus, it can be assumed that the antioxidant activity of the composite membranes was mainly due to the embedded curcumin. In particular, the collagen–curcumin and the chitosan–collagen–curcumin patches already presented the maximum radical scavenging activity after 15 min of membrane soaking in PBS (orange bars) and accounted for 19% and 28% of the activity, respectively, and did not significantly change after 60 min incubation (red bars). Conversely, the chitosan–curcumin sample showed an increasing radical scavenging activity during the studied time points and reaching 26% of the activity after 15 min and 37% after 60 min, indicating a slower release of curcumin in the PBS allowing the increase in the radical scavenging activity from 15 to 60 min. The scavenging activities of the membranes did not further increase over time since values similar to the ones measured at 60 min were also registered after 4 h of soaking (data not shown), indicating that the majority of curcumin was released during the first hour of soaking in an isotonic buffer. Among the three nanofibrous membranes, the highest radical scavenging activity was registered in the chitosan–curcumin and chitosan–collagen–curcumin mats after 15 min of soaking (orange bars) and in the chitosan–curcumin one after 60 min of soaking (red bar). Overall, these results clearly demonstrate the advantage of the use of curcumin-embedded composite membranes over membranes not containing the bioactive molecule for the beneficial anti-inflammatory effect resulting in an added value for the pharmacological use of these biomaterials.

**Figure 6:**
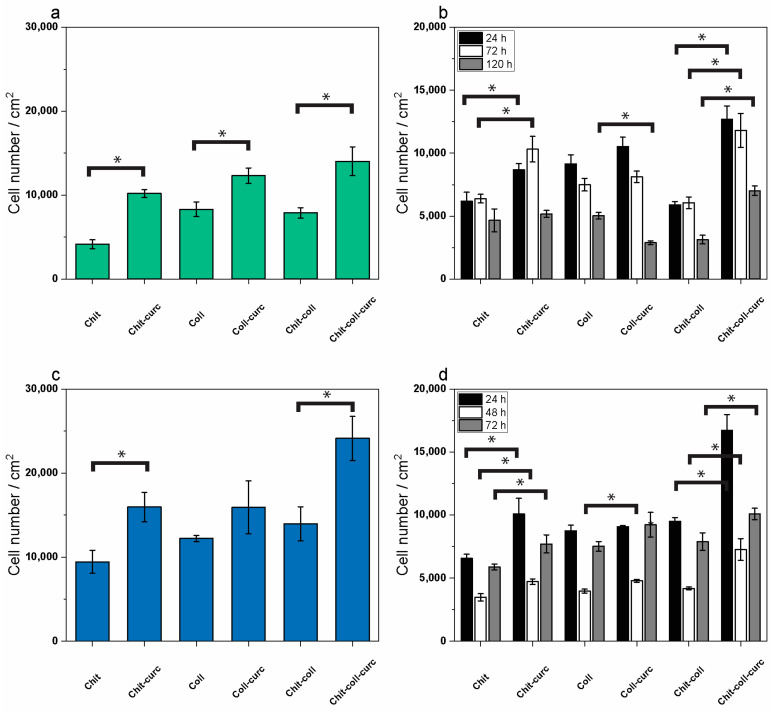
Cell adhesion and cell survival/proliferation on membranes as measured by MTT cell viability assays. (**a**) HaCaT human keratinocyte adhesion to the different membranes with or without curcumin after 16 h incubation. (**b**) HaCaT human keratinocyte survival/proliferation on the different membranes with or without curcumin after 24 h (black bars), 72 h (white bars), and 120 h (grey bars) of incubation. (**c**) L929 mouse fibroblast adhesion analyzed in the same conditions as (**a**). (**d**) L929 mouse fibroblast proliferation analyzed in the same conditions as (**b**). Results are expressed as the number of cells per cm^2^ of each membrane and are the mean ± S.D. of 3 experiments performed in quadruplicate. Statistical analysis is indicated by asterisks. Each asterisk on a bar indicates significance (* *p* < 0.005) in paired Student *t*-test between the respective membrane (i.e., collagen, chitosan, or composite) with or without curcumin.

To evaluate the biocompatibility of the composite membranes, cell adhesion and survival/proliferation of two keratinocyte and fibroblast cell lines were evaluated in time-course experiments. In particular, cell adhesion was evaluated in HaCaT human keratinocytes and L929 mouse fibroblasts by MTT tests of cells attached to the membranes after 16 h of incubation at 37 °C. The results are displayed in Figure 6a,c for HaCaT and L929 cells, respectively, showing the number of attached cells/cm^2^ to each membrane. At first sight, a higher propensity to attachment was observed for L929 fibroblasts as compared to HaCaT keratinocytes for all six membranes and, in both cell lines, the curcumin-embedded membranes seemed to be preferred as adhesion substrates. In keratinocytes (Figure 6a), the lowest number of attached cells was observed in the chitosan membranes (~7800 cells/cm^2^), while the highest number was measured in the composite chitosan–collagen–curcumin mat (~14,000 cells/cm^2^). In all cases, the addition of curcumin significantly increased the number of attached keratinocytes to the membranes. This amounts to an ~1.5-, 2.5-, and 1.8-fold increase for the collagen–curcumin, chitosan–curcumin, and chitosan–collagen–curcumin samples, respectively, as compared to the same membranes without curcumin (*p* < 0.005 for all). In fibroblasts (Figure 6c), a similar behavior was observed with the lowest number of attached cells after 16 h again measured in the chitosan membrane (~9400 cells/cm^2^) and the highest in the composite chitosan–collagen–curcumin membrane (~24,000 cells/cm^2^). Also, in this case, the presence of curcumin ameliorated cell adhesion to the mats with an increase of attached cells of ~1.3 fold for collagen–curcumin (*p* > 0.05) and of 1.7 fold for both chitosan–curcumin and chitosan–collagen–curcumin membranes as compared to the respective membranes without curcumin (*p* < 0.005 for both). Overall, the data indicate that the composite chitosan–collagen membrane with the embedded curcumin is the best substrate for cell adhesion for both fibroblasts and keratinocytes among the investigated biomaterials.

Concerning cell survival/proliferation, this feature was investigated in keratinocytes and fibroblasts in a time-course experiment at 24, 78, and 120 h; the number of cells attached to the membranes were counted by the MTT test. The results are displayed in Figure 6b,d for HaCaT and L929 and show the different behaviors of the two cell lines in the interaction with the various substrates over time. However, at the end of the experiment (120 h) again, it was possible to confirm that the curcumin-embedded membranes performed better than their respective counterparts without curcumin in ensuring cell survival/proliferation. In particular, for HaCaT keratinocytes (Figure 6b), the number of attached cells on each membrane was almost stable between 24 and 72 h (black bars and white bars, respectively), with only a slight decrease during this time in the collagen membrane and the collagen–curcumin mat, while at 120 h (grey bars) a significant decrease in attached cells was observed in all types of membranes except for the chitosan one which always maintained a similar number of cells from 24 to 120 h of incubation. Furthermore, the chitosan–curcumin membrane showed a higher number of attached cells at both 24 and 72 h as compared to the chitosan membrane alone, while the chitosan–collagen–curcumin one showed a higher number of attached cells at all time points as compared to the same membrane without curcumin. Nonetheless, at the end of the 120 h of incubation, the highest number of attached cells was measured in the composite chitosan–collagen–curcumin membrane with respect to the others. These data indicate that the keratinocytes show a tendency to detach from all six different substrates during the five-day incubation time and that curcumin addition to the chitosan–collagen composite membrane helps to slow down this tendency.

In general, in fibroblasts (Figure 6d), it was possible to observe a significant decrease in the number of attached cells to all types of membranes from 24 (black bars) to 72 h (white bars) and subsequently an increase at 120 h (grey bars) indicating that after 3 days of adaptation, the cells were able to resume proliferation in all the investigated substrates. Furthermore, the addition of curcumin to the chitosan and to the composite chitosan–collagen membranes allows for a significant increase in the number of attached cells at all time points investigated as compared to the respective membranes not embedded with the bioactive molecule. Also, for fibroblasts as well as for keratinocytes, at the end of the time course (120 h), the highest number of attached cells was measured in the composite chitosan–collagen–curcumin membrane with respect to all other membranes. These data indicate that despite requiring longer times for the adaptation of keratinocytes to the investigated substrates (i.e., collagen alone, chitosan alone, or composite chitosan–collagen), both cell types had increased attachment and survival/proliferation rates upon the addition of curcumin to the produced membranes.

### 3.4. Bacterial Adhesion Evaluation

The prevention of bacterial adhesion and biofilm formation on wound dressing devices should assist in reducing associated infections. To investigate the antibacterial behavior of the electrospun mats in terms of reduction of biofilm formation, they were incubated for 5 h in the presence of three different types of bacteria, i.e., the Gram-negative *Escherichia coli* and *Pseudomonas aeruginosa*, and the Gram-positive *Staphylococcus aureus*. The mats were added into suspensions at a cell density of 10^7^ CFU/mL and, after washing with PBS, the number of bacterial cells retained was evaluated. As shown in Table 2, comparing the level of *E. coli* cell adhesion among the three non-curcumin-embedded membranes, no significant differences were observed between chitosan-based and collagen-based electrospun mats, which had 4.05 · 10^4^ ± 1.78 · 10^4^ CFU and 1.27 · 10^4^ ± 3.78 · 10^4^ CFU, respectively. However, when the two biopolymers were combined, the antibacterial effect of chitosan results improved. After 5 h of incubation in the *E. coli* suspension, the collagen–chitosan composite mats had 4.1 · 10^3^ ± 7.5 · 10^2^ CFU. The addition of curcumin to each type of sample caused a significant decrease in *E. coli* attachment, as the bacterial cells retained results of 7.53 · 10^3^ ± 1.14 · 10^3^, 8.01 · 10^2^ ± 2.60 · 10^2^, and 2.05 · 10^3^ ± 1.70 · 10^3^ CFU for the collagen-based, chitosan-based and composite mats, respectively. Although the number of attached bacteria on the mat was 10 times higher for *E. coli*, there were no significant differences in Gram-negative *P. aeruginosa* adhesion between the non-curcumin-embedded collagen and chitosan membranes (1.55 · 10^6^ ± 4.72 · 10^5^ and 8.57 · 10^5^ ± 5.8 · 10^5^ CFU, respectively). Conversely, the combination of the two polymers significantly reduced bacterial adhesion on the composite mats. After 5 h of incubation in the *P. aeruginosa* suspension, the cells retained decreased to 1.31 · 10^4^ ± 4.83 · 10^3^ CFU. When the electrospun mats were embedded with curcumin, a significant reduction of bacterial adhesion was observed only for collagen-based mats (5.39 · 10^5^ ± 3.7 · 10^5^ CFU), while no significant differences were detected for the chitosan and composite samples. The non-curcumin-embedded composite collagen–chitosan-based and chitosan-based membranes incubated with Gram-positive *S. aureus* registered a strong reduction in cell adhesion with respect to collagen types. As shown in Table 2, there were 3.35 · 10^6^ ± 8.58 · 10^5^ CFU, 4.66 · 10^4^ ± 6.61 · 10^3^ CFU, and 3.88 · 10^4^ ± 1.73 · 10^3^ CFU attached to the collagen, chitosan, and composite samples, respectively. Finally, the addition of curcumin to the electrospun mats seemed to strongly reduce the *S. aureus* adhesion only for the collagen-based mats, which had 4.35 · 10^3^ ± 2.25 · 10^3^ CFU, while no significant differences were observed when added to chitosan-based mats. Moreover, in the composite mats, the bacterial adhesion results increased to 2.68 · 10^5^ ± 1.35 · 10^5^ CFU with respect to the non-curcumin-embedded counterpart, in which 3.88 · 10^4^ ± 1.73 · 10^3^ CFU were counted. Collagen is a very attractive biopolymer for wound-healing applications; however, many bacterial species, such as *S. aureus* and *P*. *aeruginosa*, tend to attach to collagen fibers via specific interactions [43,44]. In this study, in collagen-based electrospun mats, the *S. aureus* and *P*. *aeruginosa* cell number results were 100-fold higher than that of *E. coli* cells. A significant reduction in cell adhesion was observed in all three bacterial species tested when collagen was combined with chitosan. Although the antibacterial properties of chitosan are well documented [45], this study seems to show a better performance in bacterial adhesion reduction when combined with collagen. Compared to the collagen samples, pure chitosan-based electrospun mats showed a marked reduction in bacterial cell adhesion only with Gram-positive *S. aureus*. The different adhesion response to the electrospun mats shown by the three different bacteria species tested in this study confirm that microorganism adhesion to the biomaterials is driven by several factors such as chemical composition and ultrastructural organization of the surface. The molecular basis of the antimicrobial properties of chitosan is not yet fully known. In addition to the electrostatic interactions between the biopolymer and the bacterial membrane, many other factors could come into play, such as the polymer size and three-dimensional organization. The more homogeneous structure of the fibers, as well as their larger dimensions and the consequent reduction of the pore diameters, make the collagen–chitosan composite electrospun mats, overall, the best performing ones in terms of prevention of biofilm formation. The antibacterial properties of curcumin are widely described in different types of microorganisms [46]. Among the antimicrobial properties attributed to curcumin, there is the effect of controlling *quorum sensing* and reducing biofilm formation on surfaces. This property is extremely useful for biomaterials designed for wound dressings. Comparing the level of bacterial adhesion on the mats embedded with curcumin, it is possible to observe that, in the collagen mats, the addition of curcumin reduces the adhesion in all the species tested, and this could be due to the higher release level of the substance in a short period of time (Figure 5b). However, the level of bacterial adhesion reduction was different for the three types of bacteria, resulting in very strong reduction in *S. aureus* and weak reduction in *P. aeruginosa,* which, as previously demonstrated, is less sensitive to the antibacterial activity of curcumin [47]. While the addition of curcumin can reduce *E. coli* adhesion in all three types of polymer compositions, for *S. aureus* and *P. aeruginosa*, bacterial adhesion was not affected when curcumin was embedded in the chitosan-based mats or in the composite ones. For *S. aureus*, the marked antibacterial effect of chitosan seems to mask the beneficial effect of curcumin. Overall, considering the lower curcumin release within the bacterial incubation time when it is embedded in chitosan and composites than in collagen, the different bacterial responses to chitosan-based and composite mats embedded with curcumin suggests a lower sensitivity to curcumin’s antibiofilm effect of *S. aureus* and *P. aeruginosa* than *E. coli*. 

## 4. Conclusions

Composite chitosan–collagen electrospun membranes embedded with curcumin were prepared via a single-step electrospinning technique and explored as possible wound-healing patches by sustaining skin cell proliferation, diminishing oxidative stress, and thus inflammation, and slowing down bacterial growth. The use of non-toxic solvents and mild crosslinking conditions during the entire processing allows the preservation of the intrinsic biocompatibility of the biopolymers without affecting the nanofibrous structure and physical–chemical properties. In particular, combining chitosan and collagen leads to smooth, homogeneous nanofibers with appropriate mechanical properties and vapor permeability that are able to withstand physiological-like conditions for up to 14 days. Interestingly, along with the physical crosslinking treatment, the intermolecular interactions occurring between the macromolecular chains provide a further stabilization effect. Additionally, curcumin was found to be completely embedded within the crosslinked composite nanofibers, thus ensuring a sustained, prolonged release within 72 h when in contact with physiological fluids. All these factors were confirmed to highly affect the mat biological properties as chitosan–collagen membranes containing curcumin showed the greatest capability to foster cell viability and prevent biofilm formation. In conclusion, we demonstrated that highly porous, nanofibrous scaffolds that combine the antibacterial properties of chitosan, the affinity of collagen for living tissues, and the antioxidant properties of curcumin represent an efficient strategy for the development of the future generation of wound-healing patches. 

## Figures and Tables

**Table 1:** Water-related properties and percentage weight loss in physiological conditions for electrospun, crosslinked mats containing curcumin.

Sample	Water Contact Angle (°)	Water Vapor Permeability (g/m·Pa·s)	Moisture Content (%)	Weight Loss (%)
Chitosan–curcumin	61 ± 6	7.29 · 10^−12^	7 ± 2	97 ± 1
Collagen–curcumin	45 ± 2	7.38 · 10^−12^	6 ± 1	63 ± 3
Chitosan–collagen–curcumin	52 ± 3	4.7 · 10^−12^	6 ± 1	71 ± 2

**Table 2:** Evaluation of bacterial adhesion on different electrospun, crosslinked mats immersed for 5 h in bacterial suspension at 10^7^ CFU/mL and washed twice with sterile PBS.

Sample	*E. coli* CFU	*P. aeruginosa* CFU	*S. aureus* CFU
Chitosan	1.27 · 10^4^ ± 3.78 · 10^3^	8.57 · 10^5^ ± 5.58 · 10^5^	4.66 · 10^4^ ± 6.61 · 10^3^
Collagen	4.05 · 10^4^ ± 1.78 · 10^4^	1.55 · 10^6^ ± 4.72 · 10^5^	3.35 · 10^6^ ± 8.58 · 10^5^
Chitosan–collagen	4.17 · 10^3^ ± 7.58 · 10^2^	1.31 · 10^4^ ± 4.82 · 10^3^	3.88 · 10^4^ ± 1.73 · 10^3^
Chitosan–curcumin	8.01 · 10^2^ ± 2.60 · 10^2^	5.24 · 10^5^ ± 3.34 · 10^5^	5.21 · 10^4^ ± 1.80 · 10^4^
Collagen–curcumin	7.53 · 10^3^ ± 1.14 · 10^3^	5.39 · 10^5^ ± 3.70 · 10^5^	4.34 · 10^3^ ± 2.25 · 10^3^
Chitosan–collagen–curcumin	2.02 · 10^3^ ± 1.70 · 10^2^	8.43 · 10^4^ ± 5.71 · 10^4^	2.68 · 10^5^ ± 1.35 ·10^5^

## Data Availability

Data will be provided on request.

## Supplementary Materials

The following supporting information can be downloaded at: https://www.mdpi.com/article/10.3390/polym15132931/s1, Figure S1: Steady-state viscosity curves for (a) chitosan-based, (b) collagen-based and (c) chitosan-collagen-based solutions showing a well-defined shear-thinning behaviour. The three solutions present a viscosity in the ideal range for electrospinning. Experimental data are fitted with Carreau-Yasuda model (dashed lines) to calculate the zero-shear viscosity.; Figure S2: (a) Amplitude sweep test for chitosan-based solution showing a wide linear viscoelastic region (LVER). Frequency sweep test for (b) chitosan-based, (c) collagen-based and (d) chitosan-collagen-based solutions showing a liquid-like response (i.e., G″ > G′) in the investigated frequency range.; Figure S3: Fiber diameter distribution for (a) chitosan-based, (b) collagen-based and (c) chitosan-collagen-based electrospun mats after crossliking. Collagen mat is characterized by the smallest and least homogeneous nanofibers, whereas chitosan-collagen sample present the biggest and most regular ones.; Figure S4: TGA and DTGA profiles for (a,b) PEO powder and (c,d) curcumin powder. PEO is characterized by a well-defined, rapid degradation step occurring at T ~ 400 °C, whereas curcumin degrades in a broad temperature range of 200–400 °C.; Figure S5: DSC profiles for (a) polymer and curcumin powders, (b) pristine electrospun mats and (c) crosslinked electrospun mats. Pristine and crosslinked mats present two endothermic peaks that are associated with the melting of PEO and the evaporation of residual humidity within the samples, respectively.; Figure S6: FTIR spectra for chitosan powder (grey line), collagen powder (green line), PEO powder (purple line) and curcumin powder (orange line).; Figure S7: FTIR spectra for chitosan-curcumin pristine mat (grey line), collagen-curcumin crosslinked mat (green line) and chitosan-collagen-curcumin mat (red line). All the absorption bands of the raw materials can be observed along with slight shift in the position of amide I, II and III bands of collagen likewise due to the occurrence of a new linkage between the polymeric chains.; Figure S8: SEM micrographs after 2 weeks of immersion in PBS buffer solution (pH = 7.4, T = 37 °C) for (a) chitosan-curcumin crosslinked mat, (b) collagen-curcumin crosslinked mat and (c) chitosan-collagen-curcumin crosslinked mat. A clear disruption of the nanofibrous structure can be observed in all samples and it can be ascribed to the dissolution of the co-spinning agent (i.e., PEO) and the progressive swelling of the nanofibers.; Figure S9: Antioxidant activity of membranes. Radical scavenging activity of substances released by 15 min (orange bars) or 60 min (red bars) soaking of the membranes in PBS solution measured by the DPPH assay. Results are expressed as the percentage of scavenging activity of each membrane respect to the negative control and are the mean ± S.D. of 3 experiments performed in duplicate. Asterisks indicate significance in paired t-test vs the respective sample at 15 min soaking and vs all other samples at 60 min soaking (* *p* < 0.05).

## References

[1] Tottoli E.M., Dorati R., Genta I., Chiesa E., Pisani S., Conti B. (2020). Skin Wound Healing Process and New Emerging Technologies for Skin Wound Care and Regeneration. Pharmaceutics.

[2] Patterson C.W., Stark M., Sharma S., Mundinger G.S. (2019). Regeneration and expansion of autologous full-thickness skin through a self-propagating autologous skin graft technology. Clin. Case Rep..

[3] Mastroianni M., Ng Z.Y., Goyal R., Mallard C., Farkash E.A., Leonard D.A., Albritton A., Shanmugarajah K., Kurtz J.M., Sachs D.H. (2018). Topical Delivery of Immunosuppression to Prolong Xenogeneic and Allogeneic Split-Thickness Skin Graft Survival. J. Burn Care Res..

[4] Dong R., Guo B. (2021). Smart wound dressings for wound healing. Nano Today.

[5] Rezvani Ghomi E., Khalili S., Nouri Khorasani S., Esmaeely Neisiany R., Ramakrishna S. (2019). Wound dressings: Current advances and future directions. J. Appl. Polym. Sci..

[6] Aljghami M.E., Saboor S., Amini-Nik S. (2018). Emerging Innovative Wound Dressings. Ann. Biomed. Eng..

[7] Miguel S.P., Figueira D.R., Simões D., Ribeiro M.P., Coutinho P., Ferreira P., Correia I.J. (2018). Electrospun polymeric nanofibres as wound dressings: A review. Colloids Surf. B Biointerfaces.

[8] Rodrigues M., Kosaric N., Bonham C.A., Gurtner G.C. (2019). Wound Healing: A Cellular Perspective. Physiol. Rev..

[9] Wen P., Zong M.H., Linhardt R.J., Feng K., Wu H. (2017). Electrospinning: A novel nano-encapsulation approach for bioactive compounds. Trends Food Sci. Technol..

[10] Dodero A., Alberti S., Gaggero G., Ferretti M., Botter R., Vicini S., Castellano M. (2021). An Up-to-Date Review on Alginate Nanoparticles and Nanofibers for Biomedical and Pharmaceutical Applications. Adv. Mater. Interfaces.

[11] Keshvardoostchokami M., Majidi S.S., Huo P., Ramachandran R., Chen M., Liu B. (2020). Electrospun Nanofibers of Natural and Synthetic Polymers as Artificial Extracellular Matrix for Tissue Engineering. Nanomaterials.

[12] Soares R.M.D., Siqueira N.M., Prabhakaram M.P., Ramakrishna S. (2018). Electrospinning and electrospray of bio-based and natural polymers for biomaterials development. Mater. Sci. Eng. C.

[13] Puertas-Bartolomé M., Mora-Boza A., García-Fernández L. (2021). Emerging Biofabrication Techniques: A Review on Natural Polymers for Biomedical Applications. Polymers.

[14] Abbasian M., Massoumi B., Mohammad-Rezaei R., Samadian H., Jaymand M. (2019). Scaffolding polymeric biomaterials: Are naturally occurring biological macromolecules more appropriate for tissue engineering?. Int. J. Biol. Macromol..

[15] Wang F., Hu S., Jia Q., Zhang L. (2020). Advances in Electrospinning of Natural Biomaterials for Wound Dressing. J. Nanomater..

[16] Bayón B., Berti I.R., Gagneten A.M., Castro G.R., Singhania R.R., Agarwal R.A., Kumar R.P., Sukumaran R.K. (2018). Waste to Wealth.

[17] Wróblewska-Krepsztul J., Rydzkowski T., Michalska-Pożoga I., Thakur V.K. (2019). Biopolymers for Biomedical and Pharmaceutical Applications: Recent Advances and Overview of Alginate Electrospinning. Nanomaterials.

[18] Biswas M.C., Jony B., Nandy P.K., Chowdhury R.A., Halder S., Kumar D., Ramakrishna S., Hassan M., Ahsan M.A., Hoque M.E. (2022). Recent Advancement of Biopolymers and Their Potential Biomedical Applications. J. Polym. Environ..

[19] George A., Sanjay M.R., Srisuk R., Parameswaranpillai J., Siengchin S. (2020). A comprehensive review on chemical properties and applications of biopolymers and their composites. Int. J. Biol. Macromol..

[20] Mohebbi S., Nezhad M.N., Zarrintaj P., Jafari S.H., Gholizadeh S.S., Saeb M.R., Mozafari M. (2018). Chitosan in Biomedical Engineering: A Critical Review. Curr. Stem Cell Res. Ther..

[21] Kurakula M., Naveen N.R. (2020). Prospection of recent chitosan biomedical trends: Evidence from patent analysis (2009–2020). Int. J. Biol. Macromol..

[22] Liu X., Zheng C., Luo X., Wang X., Jiang H. (2019). Recent advances of collagen-based biomaterials: Multi-hierarchical structure, modification and biomedical applications. Mater. Sci. Eng. C.

[23] Gu L., Shan T., Ma Y.X., Tay F.R., Niu L. (2019). Novel Biomedical Applications of Crosslinked Collagen. Trends Biotechnol..

[24] Ahangari N., Kargozar S., Ghayour-Mobarhan M., Baino F., Pasdar A., Sahebkar A., Ferns G.A.A., Kim H.W., Mozafari M. (2019). Curcumin in tissue engineering: A traditional remedy for modern medicine. BioFactors.

[25] Pozzolini M., Scarfì S., Gallus L., Castellano M., Vicini S., Cortese K., Gagliani M.C., Bertolino M., Costa G., Giovine M. (2018). Production, Characterization and Biocompatibility Evaluation of Collagen Membranes Derived from Marine Sponge Chondrosia reniformis Nardo, 1847. Mar. Drugs.

[26] Dodero A., Donati I., Scarfì S., Mirata S., Alberti S., Lova P., Comoretto D., Alloisio M., Vicini S., Castellano M. (2021). Effect of sodium alginate molecular structure on electrospun membrane cell adhesion. Mater. Sci. Eng. C.

[27] Dodero A., Alloisio M., Castellano M., Vicini S. (2020). Multilayer Alginate–Polycaprolactone Electrospun Membranes as Skin Wound Patches with Drug Delivery Abilities. ACS Appl. Mater. Interfaces.

[28] Greiner A., Wendorff J.H., Heitz W., Greiner A., Wendorff J.H. (2007). Electrospinning: A Fascinating Method for the Preparation of Ultrathin Fibers. Angew. Chem. Int. Ed..

[29] Li Z., Wang C. (2013). Effects of Working Parameters on Electrospinning. Springer Briefs in Materials.

[30] Alloisio M., Dodero A., Alberti S., Vicini S., Castellano M. (2022). Electrospun alginate mats embedding silver nanoparticles with bioactive properties. Int. J. Biol. Macromol..

[31] Yasuda K., Armstrong R.C., Cohen R.E. (1981). Shear flow properties of concentrated solutions of linear and star branched polystyrenes. Rheol. Acta.

[32] Chen Z., Mo X., Qing F. (2007). Electrospinning of collagen–chitosan complex. Mater. Lett..

[33] Adamiak K., Sionkowska A. (2020). Current methods of collagen cross-linking: Review. Int. J. Biol. Macromol..

[34] Pita-López M.L., Fletes-Vargas G., Espinosa-Andrews H., Rodríguez-Rodríguez R. (2021). Physically cross-linked chitosan-based hydrogels for tissue engineering applications: A state-of-the-art review. Eur. Polym. J..

[35] Mauricio-Sánchez R.A., Salazar R., Luna-Bárcenas J.G., Mendoza-Galván A. (2018). FTIR spectroscopy studies on the spontaneous neutralization of chitosan acetate films by moisture conditioning. Vib. Spectrosc..

[36] Stani C., Vaccari L., Mitri E., Birarda G. (2020). FTIR investigation of the secondary structure of type I collagen: New insight into the amide III band. Spectrochim. Acta Part A Mol. Biomol. Spectrosc..

[37] Aziz S.B., Marif R.B., Brza M.A., Hassan A.N., Ahmad H.A., Faidhalla Y.A., Kadir M.F.Z. (2019). Structural, thermal, morphological and optical properties of PEO filled with biosynthesized Ag nanoparticles: New insights to band gap study. Results Phys..

[38] Perera K.D.C., Weragoda G.K., Haputhanthri R., Rodrigo S.K. (2021). Study of concentration dependent curcumin interaction with serum biomolecules using ATR-FTIR spectroscopy combined with Principal Component Analysis (PCA) and Partial Least Square Regression (PLS-R). Vib. Spectrosc..

[39] Andonegi M., Heras K.L., Santos-Vizcaíno E., Igartua M., Hernandez R.M., de la Caba K., Guerrero P. (2020). Structure-properties relationship of chitosan/collagen films with potential for biomedical applications. Carbohydr. Polym..

[40] Thieulin C., Pailler-Mattei C., Abdouni A., Djaghloul M., Zahouani H. (2020). Mechanical and topographical anisotropy for human skin: Ageing effect. J. Mech. Behav. Biomed. Mater..

[41] Dodero A., Vicini S., Lova P., Alloisio M., Castellano M. (2020). Nanocomposite alginate-based electrospun membranes as novel adsorbent systems. Int. J. Biol. Macromol..

[42] Gordon S. (2007). The macrophage: Past, present and future. Eur. J. Immunol..

[43] Westerlund B., Korhonen T.K. (1993). Bacterial proteins binding to the mammalian extracellular matrix. Mol. Microbiol..

[44] Switalski L.M., Patti J.M., Butcher W., Gristina A.G., Speziale P., Höök M. (1993). A collagen receptor on Staphylococcus aureus strains isolated from patients with septic arthritis mediates adhesion to cartilage. Mol. Microbiol..

[45] Yilmaz Atay H. (2020). Antibacterial activity of chitosan-based systems. Functional Chitosan: Drug Delivery and Biomedical Applications.

[46] Zheng D., Huang C., Huang H., Zhao Y., Khan M.R.U., Zhao H., Huang L. (2020). Antibacterial Mechanism of Curcumin: A Review. Chem. Biodivers..

[47] Packiavathy I.A.S.V., Priya S., Pandian S.K., Ravi A.V. (2014). Inhibition of biofilm development of uropathogens by curcumin—An anti-quorum sensing agent from Curcuma longa. Food Chem..

