# Supplementary material for: Chitosan–Collagen Electrospun Nanofibers Loaded with Curcumin as Wound-Healing Patches
Source: Polymers (Basel). 2023 Jul 2;15(13):2931. doi: 10.3390/polym15132931 (PMC10347256; doi:10.3390/polym15132931)
Supplement: Supplementary file 1 [file polymers-15-02931-s001.zip › polymers-2478277-supplementary.pdf]

Supporting Information File

Chitosan-collagen electrospun nanofibers loaded with curcumin as wound-healing patches

Maila Castellano, Andrea Dodero[1,2], Sonia Scarfi[3,4], Serena Mirata[3,4], Marina Pozzolini[3], Eleonora Tassara[3], Alina Sionkowska[5], Katarzyna Adamiak[5], Marina Alloisio[1] and Silvia Vicini[1]*

1 Department of Chemistry and Industrial Chemistry, University of Genoa, Via Dodecaneso 31, 16146 Genoa, Italy; maila.castellano@unige.it (M.C.); marina.alloisio@unige.it (M.A.); silvia.vicini@unige.it (S.V.)

2 Adolphe Merkle Institute, University of Fribourg, Chemin des Verdiers 4, 1700 Fribourg, Switzerland

3 Department of Earth, Environmental and Life Sciences, University of Genova, 16132 Genoa, Italy; soniascarfi@unige.it (S.S.); serena.mirata@edu.unige.it (S.M.); marina.pozzolini@unige.it (M.P.); eleonora.tassara@edu.unige.it (E.T.)

4 Inter-University Center for the Promotion of the 3Rs Principles in Teaching & Research (Centro 3R), 56122 Pisa, Italy

5 Department of Chemistry of Biomaterials and Cosmetics, Nicolaus Copernicus University, 87100 Toruń, Poland; alinas@umk.pl (A.S.); kadamiak@wellu.eu (K.A.)

* Correspondence: andrea.dodero@unifr.ch; Tel.: +41-26-300-9225

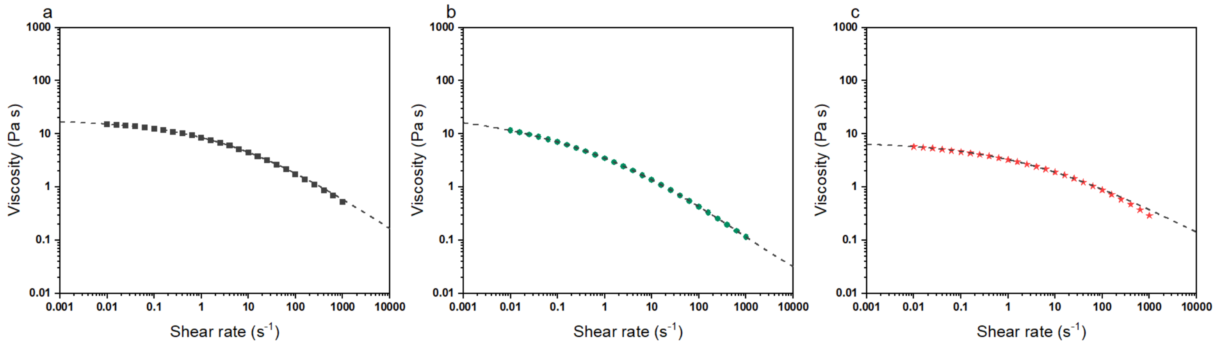

Figure S1. Steady-state viscosity curves for (a) chitosan-based, (b) collagen-based and (c) chitosan-collagen-based solutions showing a well-defined shear-thinning behaviour. The three solutions present a viscosity in the ideal range for electrospinning. Experimental data are fitted with Carreau-Yasuda model (dashed lines) to calculate the zero-shear viscosity.

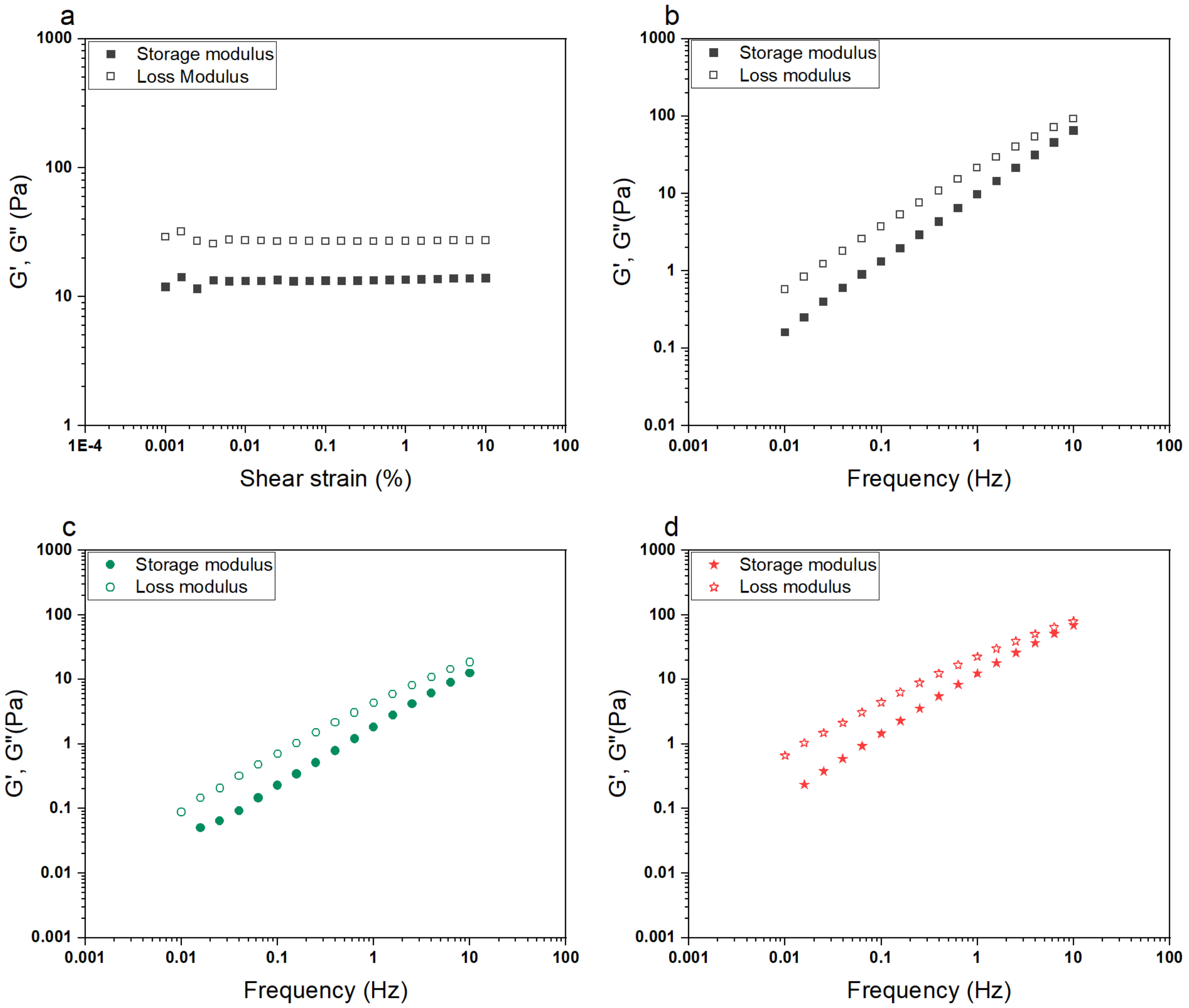

Figure S2. (a) Amplitude sweep test for chitosan-based solution showing a wide linear viscoelastic region (LVER). Frequency sweep test for (b) chitosan-based, (c) collagen-based and (d) chitosan-collagen-based solutions showing a liquid-like response (i.e., G" > G') in the investigated frequency range.

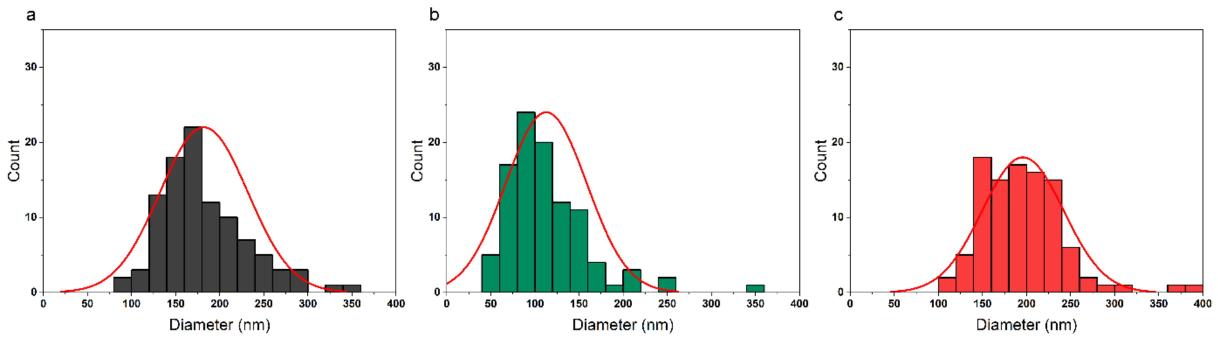

Figure S3. Fiber diameter distribution for (a) chitosan-based, (b) collagen-based and (c) chitosan-collagen-based electrospun mats after crossliking. Collagen mat is characterized by the smallest and least homogeneous nanofibers, whereas chitosan-collagen sample present the biggest and most regular ones.

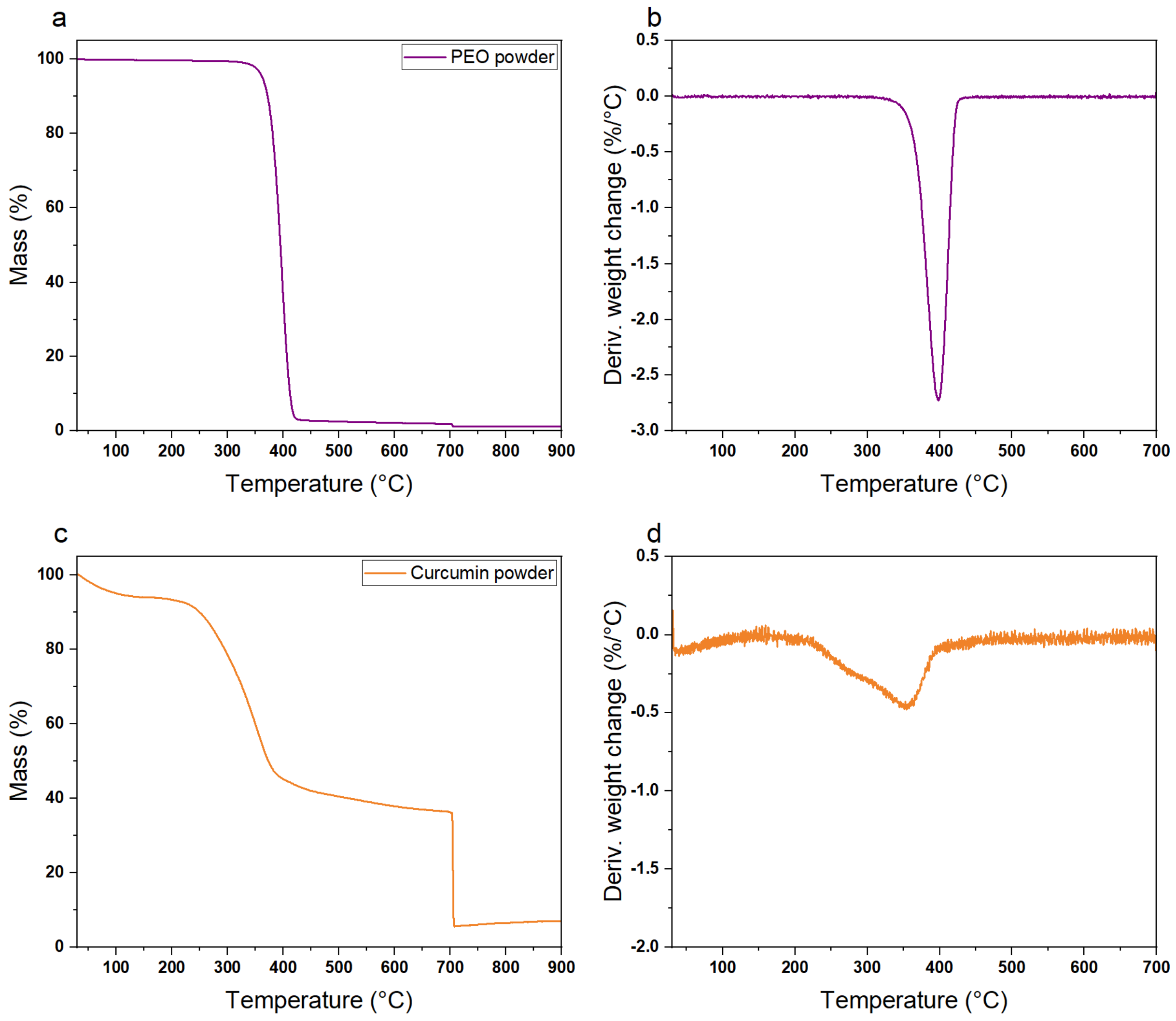

Figure S4. TGA and DTGA profiles for (a, b) PEO powder and (c, d) curcumin powder. PEO is characterized by a well-defined, rapid degradation step occurring at T ~ 400 °C, whereas curcumin degrades in a broad temperature range of 200-400 °C.

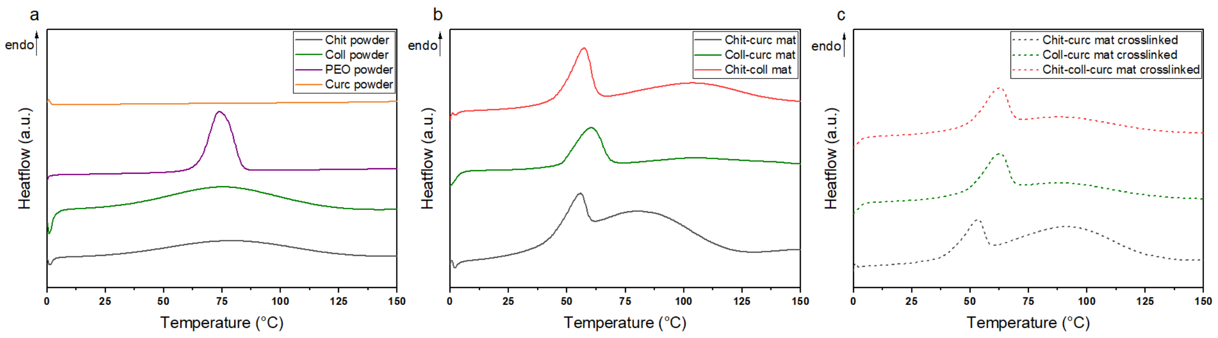

Figure S5. DSC profiles for (a) polymer and curcumin powders, (b) pristine electrospun mats and (c) crosslinked electrospun mats. Pristine and crosslinked mats present two endothermic peaks that are associated with the melting of PEO and the evaporation of residual humidity within the samples, respectively.

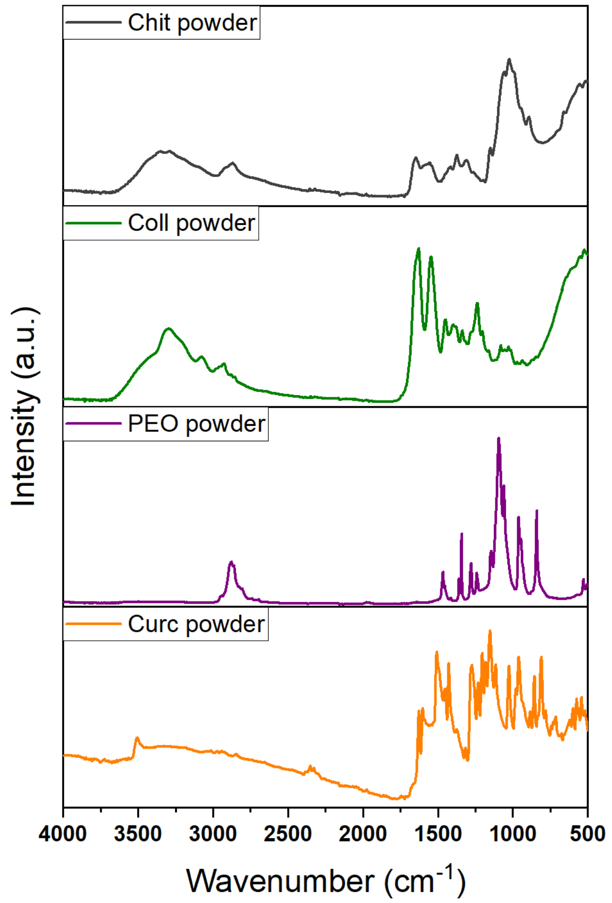

Figure S6. FTIR spectra for chitosan powder (grey line), collagen powder (green line), PEO powder (purple line) and curcumin powder (orange line).

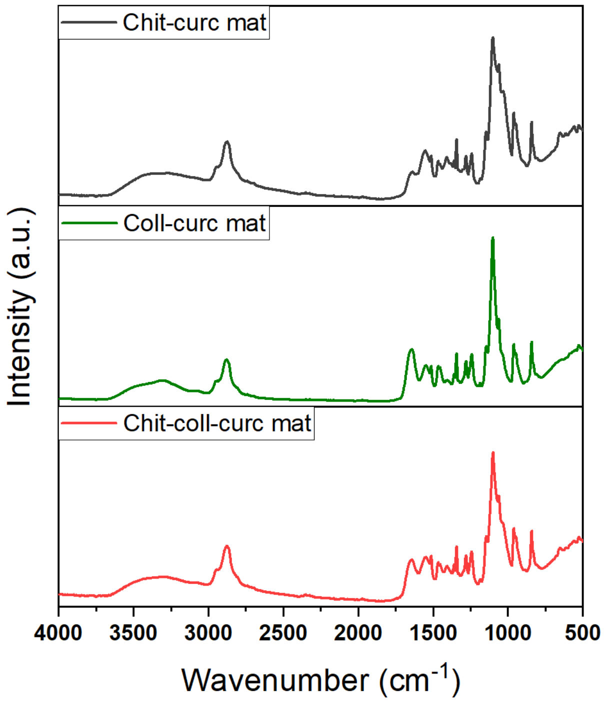

Figure S7. FTIR spectra for chitosan-curcumin pristine mat (grey line), collagen-curcumin crosslinked mat (green line) and chitosan-collagen-curcumin mat (red line). All the absorption bands of the raw materials can be observed along with slight shift in the position of amide I, II and III bands of collagen likewise due to the occurrence of a new linkage between the polymeric chains.

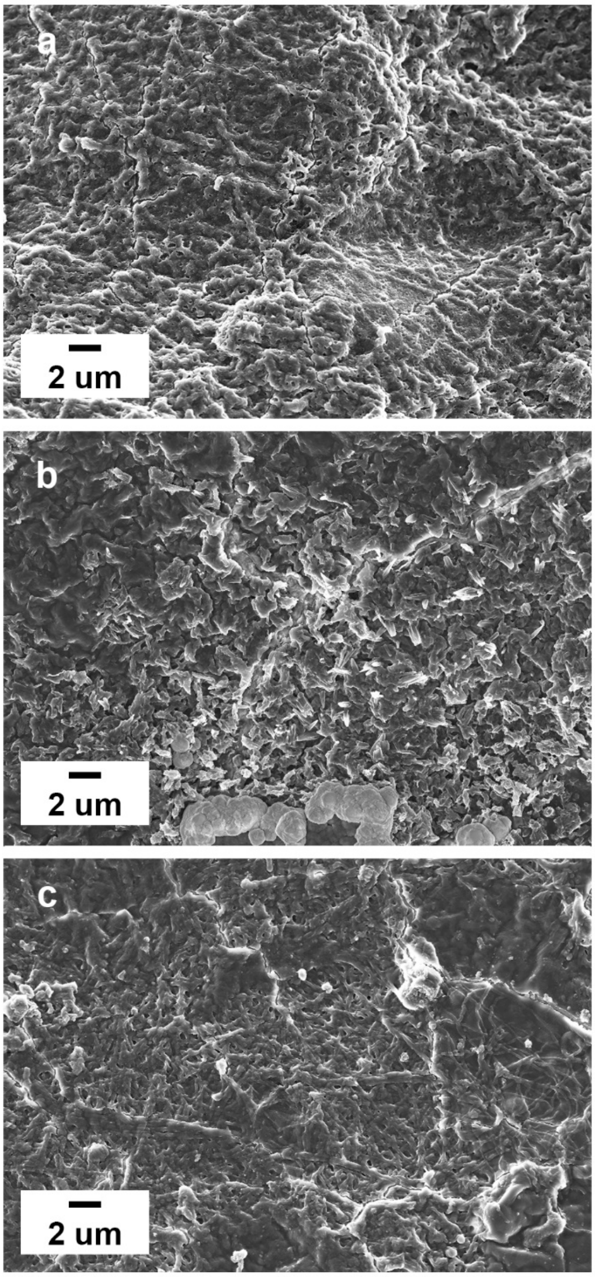

Figure S8. SEM micrographs after 2 weeks of immersion in PBS buffer solution (pH = 7.4, T = 37 °C) for (a) chitosan-curcumin crosslinked mat, (b) collagen-curcumin crosslinked mat and (c) chitosan-collagen-curcumin crosslinked mat. A clear disruption of the nanofibrous structure can be observed in all samples and it can be ascribed to the dissolution of the co-spinning agent (i.e., PEO) and the progressive swelling of the nanofibers.

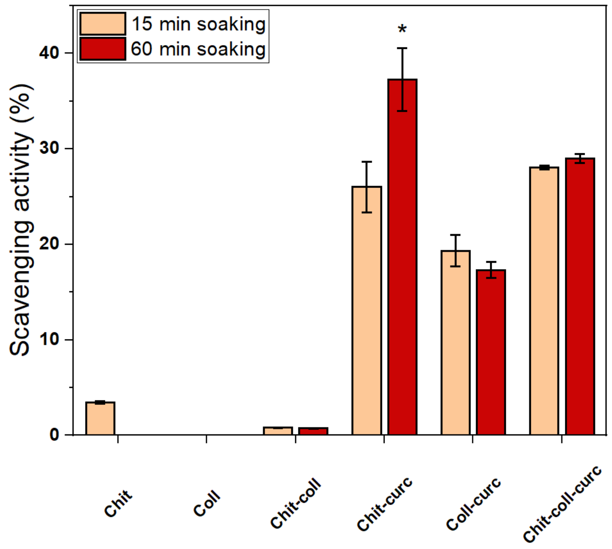

Figure S9. Antioxidant activity of membranes. Radical scavenging activity of substances released by 15 min (orange bars) or 60 min (red bars) soaking of the membranes in PBS solution measured by the DPPH assay. Results are expressed as the percentage of scavenging activity of each membrane respect to the negative control and are the mean ± S.D. of 3 experiments performed in duplicate. Asterisks indicate significance in paired t-test vs the respective sample at 15 min soaking and vs all other samples at 60 min soaking (*$p < 0.05$).
